# ﻿Description of the male of *Erromyrma* Bolton & Fisher, 2016 (Hymenoptera, Formicidae)

**DOI:** 10.3897/zookeys.1163.95696

**Published:** 2023-05-19

**Authors:** Manoa M. Ramamonjisoa, Nicole Rasoamanana, Brian L. Fisher

**Affiliations:** 1 Madagascar Biodiversity Center, BP 6257, Parc Botanique et Zoologique de Tsimbazaza, Antananarivo, Madagascar Madagascar Biodiversity Center Antananarivo Madagascar; 2 Entomology, California Academy of Sciences, 55 Music Concourse Drive, San Francisco, CA 94118, USA California Academy of Sciences San Francisco United States of America

**Keywords:** *Erromyrma*, Madagascar, male ants, morphology, Myrmicinae, Solenopsidini

## Abstract

The male of the myrmicine genus *Erromyrma* is described for the first time on the basis of two specimens of *Erromyrmalatinodis* (Mayr, 1872) collected in northern Madagascar. We used COI barcoding to confirm the identification of the male specimens as conspecific with *Erromyrmalatinodis*. We provide an illustrated male-based key to the four Myrmicinae tribes (Attini, Crematogastrini, Solenopsidini, Stenammini) and to the Solenopsidini genera (*Adelomyrmex*, *Erromyrma*, *Solenopsis*, *Syllophopsis* and *Monomorium*) for the Malagasy region.

## ﻿Introduction

Within the Malagasy region, Myrmicinae is one of the largest and most diverse subfamilies of Formicidae (Hymenoptera), with 30 genera in four tribes (Fisher and Peeters 2019; Fisher 2022). The genus *Erromyrma* Bolton & Fisher, 2016 (Solenopsidini), is represented by one species in the Malagasy region, *Erromyrmalatinodis* (Mayr, 1872). The species has been introduced in many countries, including the Malagasy region, and is thought to have originated in India (Sharaf et al. 2018). The global distribution also includes Indomalaya, the Southeastern Palearctic and Oceania bioregions. *Erromyrmalatinodis* was originally placed in *Monomorium* but was shown to be a distinct lineage within the Solenopsidini based on molecular phylogenetic evidence (Ward et al. 2015) and placed in the newly described genus *Erromyrma*. Here we present the first description of the previously unknown male of *Erromyrma* based on *E.latinodis*, collected in northern Madagascar. We provide a male-based key to the Myrmicinae tribes and to genera for the tribe Solenopsidini of the Malagasy region.

## ﻿Material and methods

This study is based on two male ant specimens (unique specimen identifiers: CASENT0788835 and CASENT0801166) collected in northern Madagascar in the town of Antsohihy (-14.89385, 47.98261) in the Region of Sofia, at c. 11 m above sea level on April 23, 2017, by Brian L. Fisher and the Madagascar Biodiversity Center team (Team Vitsika). Two males along with workers and queens were collected by hand under the bark of a mango tree along a dirt road 1 km outside of the town of Antsohihy (collection code identifiers: BLF40204, BLF40205). The mango tree was 1.5 m in diameter and approximately 5 m tall. The ants were found under bark flakes before the first branch at about 1 m in height.

Terminology for general morphology follows Bolton (1994) and Boudinot (2013, 2015). The terminology of the wing venation follows Yoshimura and Fisher (2007). When referring to the presence or absence of veins in the descriptions, a vein is considered present regardless of whether it is tubular, nebulous, or spectral (Mason 1986).

### ﻿Imaging

Digital color montage images were created using a JVC KY-F75 digital camera and Syncroscopy Auto-Montage software (ver. 5.0), or a Leica DFC 425 camera in combination with the Leica Application Suite software (ver. 3.8). These images are available online through AntWeb.org (2022) and are accessible using the unique specimen identifier code.

### ﻿Mapping

The distribution map was generated by importing specimen distribution records into the Diva-GIS program (Hijmans et al. 2011).

### ﻿Morphological study

**Figure 1.**
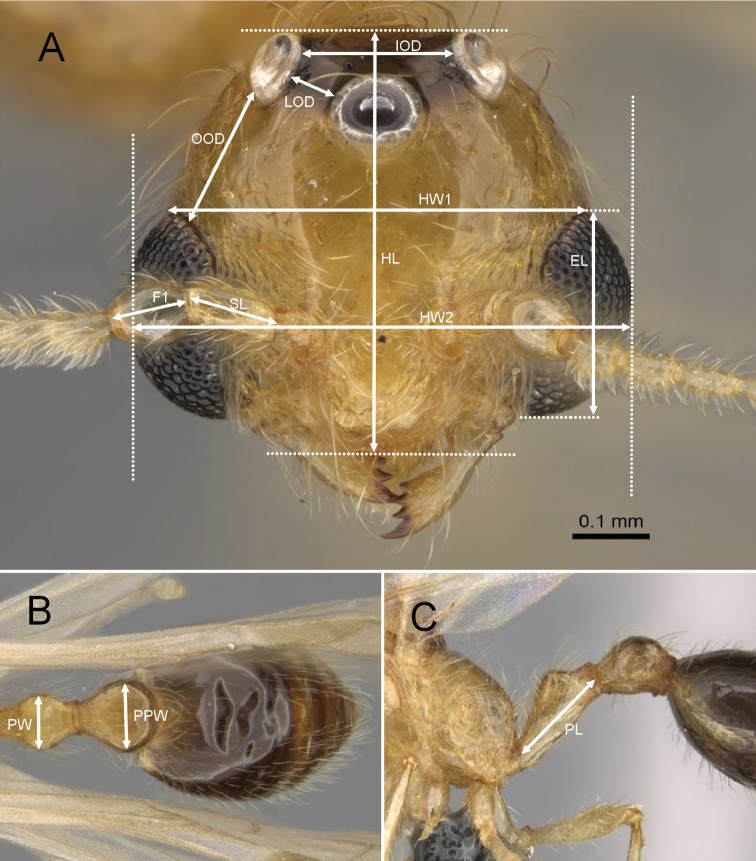
Illustration of measurements of *Erromyrmalatinodis* (CASENT0788835) **A** head in full-face view **B** segment abdominal in dorsal view **C** segment abdominal in lateral view.

Morphological observations and measurements were carried out under Leica stereoscopic microscopes (MZ9.5). All measurements (see Fig. 1) and indices are expressed in millimeters.

The following characters were recorded:

**EL**: Maximum eye length measured in full-face view along its maximum vertical diameter.

**F1**: Maximum length of the pedicel (1^st^ funicular segment, 2^nd^ antennal segment) measured in a straight line.

**HL**: Maximum head length in full-face view, measured in a straight line, from the anterior clypeal margin to the midpoint of a straight line drawn across the occipital margin.

**HW1**: Head width at the level of the posterior margin of compound eyes, measured in full-face view.

**HW2**: Maximum head width including the compound eyes, measured in full-face view.

**IOD**: Inner ocellar distance. Minimum distance between the inner edges of the two lateral ocelli, measured in full-face view.

**LOD**: Lateral ocellar distance. Minimum distance between the inner edge of the median and lateral ocelli, measured in full-face view.

**OOD**: Ocular-ocellar distance. Minimum distance from the outer edge of a lateral ocellus to the compound eyes, measured in full-face view.

**PL**: Petiole length, measured in profile view from the anterior margin of the peduncle to posterior most point of the petiolar tergite.

**PW**: Petiolar width. Maximum petiole width, measured in dorsal view.

**PPW**: Postpetiolar width. Maximum postpetiole width, measured in dorsal view.

**SL**: Scape length. Maximum length of the antennal scape measured in a straight line, excluding the basal constriction and condylar bulb.

### ﻿Indices

**CI**: Cephalic index. HW1/HL × 100.

**SI**: Scape index. SL/HW1 × 100.

**EI**: Eye index. EL/HW1 × 100.

**PI**: Petiolar index. PL/PPL.

### ﻿DNA sampling

After searching for the males in colonies across Madagascar for six years, we wanted to confirm that these males did represent the first males of *E.latinodis* even though they were collected along with queens and workers. We sequenced 658 base pairs (bp) of mitochondrial cytochrome oxidase I (COI) gene from one of the males to evaluate similarity with CO1 sequenced from 33 workers of *E.latinodis* across the region. The distribution of the specimens sequenced is shown in Fig. 10. DNA extraction and COI sequencing were performed at University of Guelph (Ontario, Canada), following the protocol described in Fisher and Smith (2008).

Abbreviation of depositories:

**BMNH** British Museum of Natural History, London, UK;

**CASC**California Academy of Sciences, San Francisco, CA, USA;

**MCZ**Museum of Comparative Zoology Cambridge, MA, USA;

**MHNG**Muséum d’histoire naturelle, Genève, Switzerland;

**MSNG**Museo Civico di Storia Naturale 'Giacomo Doria', Genova, Italy;

**NHMW**Naturhistorisches Museum, Wien (= Vienna), Austria.

## ﻿Results

The 34 specimens sequenced (see Table 1) had a within-species sequence divergence of 0.00%. Thus, based on CO1, the male specimen sequenced is conspecific with the workers from throughout the region.

**Table 1.** *Erromyrmalatinodis* Specimens sequenced for mitochondrial cytochrome oxidase I (COI) gene, including Genbank accession number, and caste. All voucher specimens are housed at the California Academy of Sciences.

Specimen Identifier	Collection Event identifier	BOLD Process ID	COI-5P GenBank	sequence length	Country	Caste
CASENT0010900-D01	R.J.1.765	ASANR501-09	HQ925412	590	Mayotte	worker
CASENT0107528-D01	BLF11668	JDWAM495-05	OP442963	654	Madagascar	worker
CASENT0107541-D01	BLF11664	JDWAM503-05	OP442956	654	Madagascar	worker
CASENT0123018-D01	BLF16532	ASANP672-09	GU710443	596	Madagascar	worker
CASENT0123025-D01	BLF16539	ASANP673-09	GU710442	625	Madagascar	worker
CASENT0123498-D01	BLF16507	ASANP676-09	HQ925385	618	Madagascar	worker
CASENT0132440-D01	BLF18832	ASANO176-09	GU709833	658	Mayotte	worker
CASENT0134112-D01	BLF19142	ASANP692-09	GU710444	658	Madagascar	worker
CASENT0134329-D01	BLF19879	ASANO717-09	GU709835	658	Comoros	worker
CASENT0134937	BLF18804	ASIMB817-09	OP442961	654	Mayotte	worker
CASENT0134955	BLF18810	ASIMB824-09	OP442957	654	Mayotte	worker
CASENT0134970	BLF18809	ASIMB832-09	OP442962	654	Mayotte	worker
CASENT0136510-D01	BLF19801	ASANO766-09	GU709838	658	Comoros	worker
CASENT0136519-D01	BLF19811	ASANO769-09	GU709837	658	Comoros	worker
CASENT0136656-D01	BLF19846	ASANO786-09	GU709840	658	Comoros	worker
CASENT0136764-D01	BLF19700	ASANO809-09	GU709839	658	Comoros	worker
CASENT0136784	BLF18809	ASIMB886-09	OP442959	654	Mayotte	worker
CASENT0136900-D01	BLF20364	ASANR766-09	GU711159	658	Madagascar	worker
CASENT0136902-D01	BLF20384	ASANP695-09	GU710446	658	Madagascar	queen
CASENT0136903-D01	BLF20384	ASANP696-09	GU710445	645	Madagascar	worker
CASENT0137058-D01	BLF19947	ASANO840-09	GU709842	658	Comoros	worker
CASENT0137059-D01	BLF19947	ASANO841-09	GU709841	658	Comoros	worker
CASENT0137334-D01	BLF19951	ASANO912-09	GU709844	658	Comoros	worker
CASENT0137487-D01	BLF19767	ASANO967-09	GU709836	658	Comoros	worker
CASENT0145999-D01	BLF21147	ASANQ049-09	GU710903	658	Comoros	worker
CASENT0146463-D01	BLF21164	ASANQ138-09	GU710902	658	Comoros	worker
CASENT0146468-D01	BLF21187	ASANQ140-09	GU710905	658	Comoros	worker
CASENT0146475-D01	BLF21160	ASANQ144-09	GU710904	658	Comoros	worker
CASENT0146479-D01	BLF21176	ASANQ146-09	GU710907	658	Comoros	worker
CASENT0146495-D01	BLF21188	ASANQ150-09	GU710906	658	Comoros	worker
CASENT0147204-D01	BLF20835	ASANQ268-09	GU710909	658	Comoros	worker
CASENT0189653	BLF18804	ASIMB946-09	OP442955	654	Mayotte	worker
CASENT0189654	BLF18810	ASIMB947-09	OP442960	654	Mayotte	worker
CASENT0788835-D01	BLF40204	BFANT381-22	OP442958	658	Madagascar	male

### ﻿Taxonomic synopsis

#### ﻿ Erromyrma latinodis

Taxon classificationAnimaliaHymenopteraFormicidae

(Mayr, 1872)

36715004-E978-527A-806B-D29EF96D47B1

Monomorium latinode Mayr, 1872: 152 (w.). Lectotype worker (designated by Heterick 2006: 108): Malaysia (“Borneo”), Sarawak, 1865–66 (J. Doria & O. Beccari), unique specimen identifier: CASENT0010941, examined [BMNH]. Paralectotype with same data as lectotype, unique specimen identifier: CASENT0905756, examined [MSNG]. [Combination in Erromyrma: Fisher and Bolton 2016: 276].
Monomorium latinode var. bruneum Emery, 1893: 243 (w.). Lectotype worker (designated by Heterick 2006: 108): Sri Lanka (“Ceylon”), Kandy, i.–ii.1892 (E. Simon), unique specimen identifier: CASENT0008632, examined [MSNG]. [Junior synonym of latinode: Heterick 2006: 108].
Monomorium latinode var. voeltzkowi Forel, 1907: 78 (w.). Lectotype worker (designated by Heterick 2006: 108): Tanzania (“Ostafrika”), Pemba I., Chake-Chake (A. Voeltzkow) [MCZC]. Paralectotype with same data as lectotype, unique specimen identifier: CASENT0101928, examined [MHNG]. [Junior synonym of latinodis: Bolton 1987: 429].
Monomorium latinodoides Wheeler, 1928: 17 (w.). Syntype worker: China: Hong Kong, Kowloon (F. Silvestri) unique specimen identifier: MCZ-ENT00727982, examined [MCZC]. comb nov., syn. n.

##### Note.

The type series at MCZ was examined. The syntypes series are labeled “Kowloon” (F. Silvestri): one pin with 3 workers (MCZ-ENT00020883) and 2 workers and one dealate queen on a second pin (MCZ-ENT00727982). The workers match the description and diagnosis (see below) of worker of *E.latinodis*. We formally combine the species in *Erromyrma* and synonymize the species with *E.latinodis*. However, we exclude the queen (on MCZ-ENT00727982) from the syntype series; it belongs to the genus *Carebara* (Westwood, 1840).

##### Diagnosis.

***Erromyrma* workers [modified from Heterick (2006) and Fisher and Bolton (2016)**]:

1. Worker caste polymorphic
2. Palp formula 3,3
3. Mandible triangular, smooth, and shiny
4. Five mandibular teeth
5. Antenna with 12 segments, with 3-segmented apical club
6. Scape short, failing to reach occipital margin
7. Compound eyes present and conspicuous
8. Clypeus with a distinct unpaired seta at the midpoint of the anterior margin
9. Frontal carinae short and parallel
10. Antennal scrobe absent
11. Head without raised nuchal (= occipital) carina
12. Tibial spurs absent from meso- and metatibia
13. Promesonotal suture not distinct in dorsal view
14. Metanotal groove present
15. Propodeum unarmed
16. Propodeal dorsum with strong transverse striolae
17. Petiole pedunculated
18. Subpetiolar process absent
19. Sting developed

***Erromyrmalatinodis* males**:

The following combination of characters diagnose males of *Erromyrmalatinodis*.

**Figure 2.**
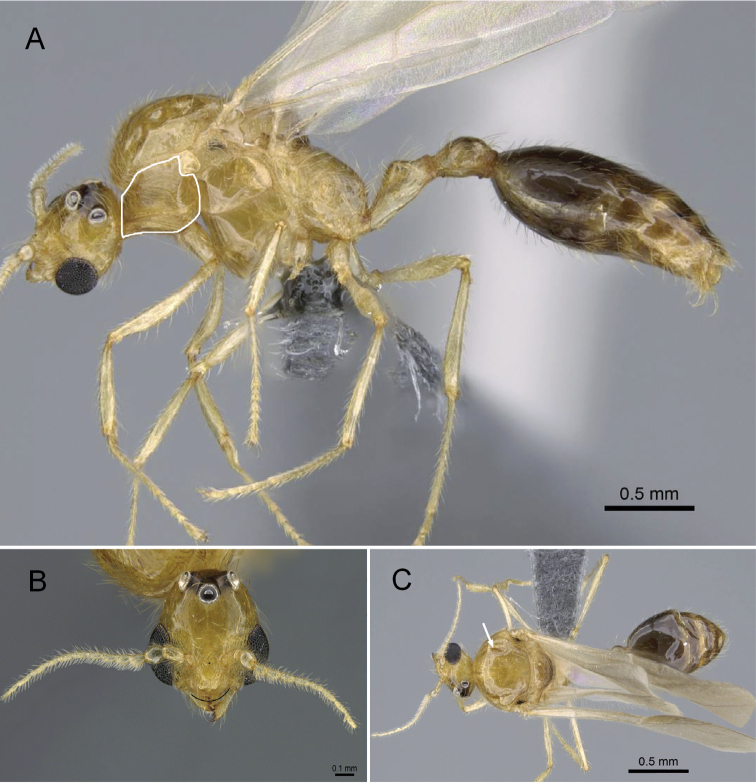
*Erromyrmalatinodis* (CASENT0788835) **A** body in lateral view **B** head in full-face view **C** body in dorsal view.

**Figure 3.**
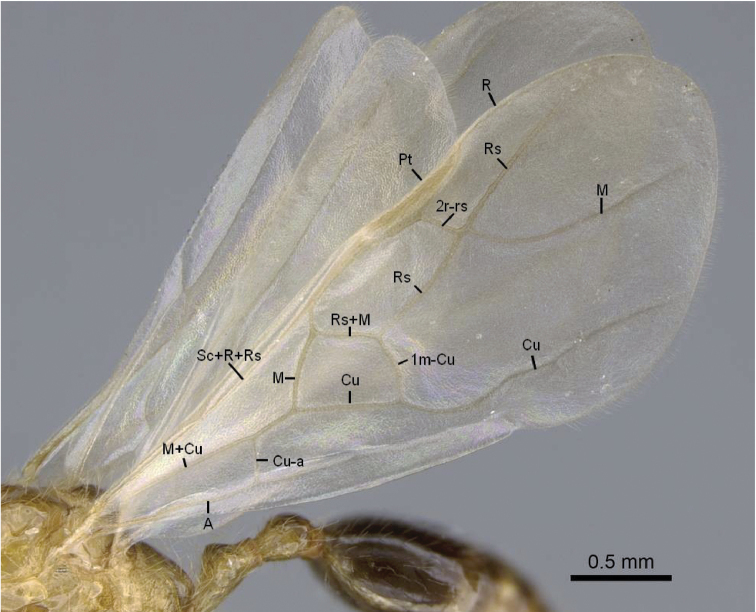
Male forewing veins of *Erromyrmalatinodis* (CASENT0788835). Abbreviations: **Pt** pterostigma; **Sc** subcosta; **R**, **r** radius; **Rs**, **rs** radial sector; **M**, **m** media; **Cu** cubitus; **A**, **a**, anal.

1. Palp formula 5,3
2. Mandible triangular with distinct basal and masticatory margins (Fig. 2B)
3. Four mandibular teeth (Fig. 2B)
4. Antenna short and filiform, with 13 segments; pedicel subglobular (Fig. 2B)
5. Scape short (SI < 33, Fig. 2B)
6. Compound eyes large (EL/HW1 0.58, Fig. 1A)
7. Ocelli present and similar in size (Fig. 1A)
8. Clypeus with anterior margin convex. (Fig. 2B)
9. Frontal carinae absent (Fig. 2B)
10. Antennal scrobe absent (Fig. 2B)
11. Head without raised nuchal (= occipital) carina (Fig. 2C)
12. Notauli absent (Fig. 2C)
13. Single spur present on meso- and metatibia (Fig. 2A)
14. Pterostigma present on the forewing (Fig. 3)
15. First median-cubital cross-vein (1m-cu) present on the forewing (Fig. 3)
16. Cubital vein of the forewing fused with the median vein (M+Cu) and forms an angle higher than 45° with M (Fig. 3)
17. Forewing cross-vein 2rs-m absent (Fig. 3)
18. Petiole pedunculate (Fig. 2A)
19. Postpetiole elongated anteriorly, subglobose in lateral view (Fig. 2A)
20. Abdominal segment IV elongate and not shouldered (Fig. 2A)
21. Pygostyles present (Fig. 2A)

Male measurements (*N* = 2). HL 0.60–0.62, HW1 0.48–0.5, HW2 0.62–0.65, EL 0.28–0.29, EW 0.21–0.23, IOD 0.20–0.21, LOD 0.06–0.07, OOD 0.16–0.18, SL 0.12–0.14, F1 0.09–0.10, PL 0.51–0.54, PW 0.20–0.21, PPW 0.33–0.34, CI 81–82, SI 28, EI 0.58.

##### Description.

***Erromyrmalatinodis* males**:

***Structure.*** In full-face view, the head including compound eyes slightly wider than long. Posterior head margin relatively rounded; head with three large, rounded ocelli of the same size, situated on the frontal face of the head, lateral ocelli directed 45° toward lateral sides. Compound eye large, strongly bulging. Anterior margin of clypeus convex. Mandible well developed and elongate triangular; masticatory margin with four teeth, the basal and masticatory margins are distinct.

Antennae 13-segmented short and filiform, pedicel subglobular. The mesoscutum is strongly convex and bulging, in the dorsal view much broader than the head at the level of the forewing insertion. In dorsal view, pronotum short in the median portion; notauli absent on the mesoscutum; mesoscutellum broader than long and smaller than mesoscutum. Metapleural gland bulla present with metapleural lobe closed. In profile, propodeal angle rounded, without spines or teeth. Hind femora longer than tibia.

In lateral view, petiole distinctly pedunculated; subpetiolar process absent (Fig. 2A). In dorsal view, abdominal segment III (postpetiole) elongated anteriorly; abdominal segment IV not shoulder and broader than the remaining tergites.

***Sculpture.*** Clypeus, dorsum, lateral face, and venter of head weakly smooth and shiny. Pro- and mesothorax extensively smooth or very superficially sculptured and shiny, with posterolateral area of mesoscutum and posterior zone of mesopleuron unsculptured with shiny area. Metanotum and metapleuron unsculptured and matte. Apical area of anterior slope of petiole, coxae, femora, and tibiae of all legs smooth to superficially sculptured and shiny; tarsi entirely microsculptured. Gaster (abdominal segments IV to the apex) entirely smooth to superficially sculptured and shiny.

***Color.*** Body and mandible largely brownish yellow except the ocellar region and the abdominal segments IV to the apex, brown.

***Pilosity.*** Anterior margin of clypeus with a pair of stout setae and without a distinct unpaired seta at its midpoint. Mandible covered with standing hairs. Antennal scape and pedicel with short and decumbent whitish hairs; the flagellomeres densely hairy. Hairs on head and body moderately abundant, erect, short, and stout. Pronotum, mesoscutum, and mesoscutellum with many obliquely standing hairs; hairs on mesopleuron much sparser; metanotum and propodeum with erect hair. Femora and tibiae with appressed hairs; tarsi covered with short appressed hairs. Posterior margins of each abdominal tergite and sternite with long and suberect hairs. Parameres covered with stout hair.

***Wings.*** (Fig. 3) Forewing with four closed cells. Costal vein (C) absent. Pterostigma pigmented, visible on the leading edge of the forewing. Radial vein (R) fused proximally to constitute Sc+R+Rs before reaching the pterostigma.

Radial sector (Rs). Past the separation from Sc+R+Rs, Rs usually short free abscissa down curved and never reaching to the costal margin, the radial sector connects to the pterostigma via the second radial-radial sector cross-vein (2r-rs). Then merging with median vein (M) and continuing fused (Rs+M).

Median vein (M). Further away from the leading wing margin is the median vein, proximally fused with cubital vein (M+Cu), following separation continuing as a free abscissa M before joining with radial sector to form Rs+M. Median vein (M) is fused with radial sector and present in past the junction of the radial sector.

Cubital vein (Cu). Proximally the cubital vein is fused with median vein (M+Cu), the cubital vein (Cu) divided by median-cubital cross-vein (1m-cu) the cubital vein does not connect to the distal wing margin.

Anal vein (A). A longitudinal vein running near the posterior wing margin. Consists of a free abscissa fused to cubital-anal cross-vein (cu-a), and continuing past cu-a.

##### Comments.

The tribe Solenopsidini is separated from other Malagasy myrmicine tribes by the following combination of characters: with the head in full-face view, mandibles with masticatory margin less than five teeth; antennal scrobe reduced to absent; pedicel not more elongated than the remaining segments; ocelli present and same size situated on the frontal face of the head, lateral ocelli directed toward oblique front sides; occipital carina not visible in full-face view; head (including compound eyes) slightly wider than long with occipital margin of head rounded. In lateral view, the anterodorsal margin of mesopleuron lower than the highest point of the wing process, pronotum and mesonotum from a smooth convexity, pronotal furrow less marked; forewing venation: cross-vein 2rs-m absent, costal vein absent, radial sector down curved and never reaching to the costal margin; propodeal spines absent; pygostyle present; abdominal segment III attached anteriorly to abdominal segment IV; peduncle of abdominal segment III is distinctly longer than that of the petiole; single tibial spur present on the front leg. In dorsal view, notauli absent.

*Erromyrma* can be distinguished from three other genera, *Adelomyrmex* (Emery, 1897), *Monomorium* (Mayr, 1855) and *Syllophopsis* (Santschi, 1915), by its subglobular pedicels. It can be separated from the genus *Solenopsis* (Westwood, 1840) by the number of its antennal segment.

### ﻿Key to the tribes of subfamily Myrmicinae based on males in the Malagasy region

The subfamily of Myrmicinae is represented by four tribes in the Malagasy region: Attini, Crematogastrini, Solenopsidini, Stenammini.

**Attini**: *Cyphomyrmex* (introduced), *Eurhopalothrix*, *Pheidole*, *Pilotrochus*, *Strumigenys*.

**Crematogastrini**: *Calyptomyrmex*, *Cardiocondyla*, *Carebara*, *Cataulacus*, *Crematogaster*, *Dicroaspis*, *Eutetramorium*, *Malagidris*, *Melissotarsus*, *Meranoplus*, *Metapone*, *Nesomyrmex*, *Pristomyrmex*, *Royidris*, *Terataner*, *Tetramorium*, *Trichomyrmex*, *Vitsika*, *Vollenhovia* (introduced?).

**Solenopsidini**: *Adelomyrmex*, *Erromyrma* (introduced), *Monomorium*, *Solenopsis*, *Syllophopsis*.

**Stenammini**: *Aphaenogaster*.

**Table:** 

1	In profile, occipital carina strongly developed (Fig. 4A); mesoscutellum strongly elevated above metanotum; in dorsal view, scutellum smooth and convex (Fig. 4C); petiole distinctly pedunculate. With the head in full-face view, mandible always triangular	**Stenammini (*Aphaenogaster*)**
–	In profile, occipital carina not forming a sharp ridge (Fig. 4B); mesoscutellum slightly convex to flat; in dorsal view, scutellum with or without sculptured (Fig. 4D); petiole sessile to shortly pedunculate. With the head in full-face view, the mandible broadly triangular to reduce	**2**
2	In profile, posterodorsal margin of head almost straight from the base of the lateral ocelli to the midpoint of the occipital carina. (Fig. 5A)	**Attini (part)**
–	In profile, posterodorsal margin of head gradually rounded from the base of the lateral ocelli to the midpoint of the occipital margin. (Fig. 5B)	**3**
3	Cross-vein 2rs-m present on forewing (Fig. 6A)	**(Attini) *Pheidole***
–	Cross-vein 2rs-m absent on forewing (Fig. 6B)	**4**
4	Mandible strongly developed; masticatory margin with 7 large teeth which increase in size from apex to base; between each tooth is a minute denticle (Fig. 7A)	**(Attini) *Pilotrochus***
–	Mandible normal to reduced; edentate to multidentate with many acute teeth which decrease in size from apex to base; without denticle between the teeth (Fig. 7B)	**5**
5	In lateral view, anterior margin of promesonotum forms a continuous outline, pronotal furrow not breaking outline (Fig. 8A)	**Solenopsidini**
–	In lateral view, anterior margin of promesonotum interrupted by an impressed pronotal furrow that breaks the outline (Fig. 8B) or mesonotum strongly produced anterodorsally (Fig. 8C)	**Crematogastrinii**

**Figure 4.**
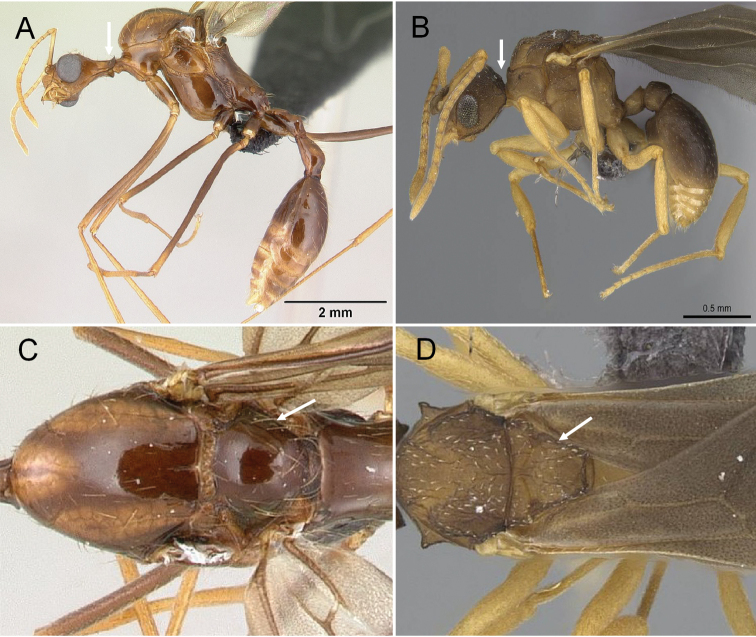
In profile view showing occipital carina **A, B***Aphaenogasterbressleri* (CASENT0495103). In dorsal view form mesoscutellum **C, D***Cyphomyrmexminitus* (CASENT0264488).

**Figure 5.**
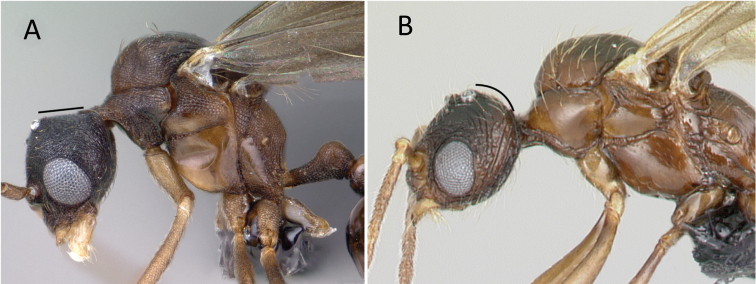
Head in profile view **A***Strumigenyschilo* (CASENT0145240) **B***Tetramoriumsilvicola* (CASENT0494732).

**Figure 6.**
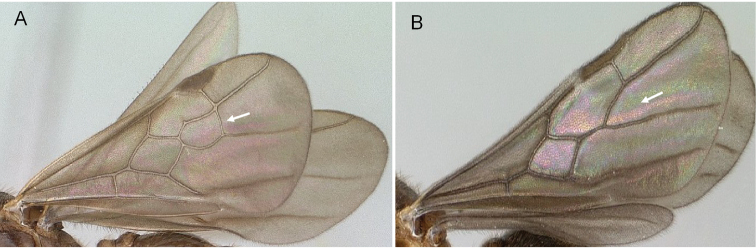
Forewing **A***Pheidole* mgs006 (CASENT0135889) **B***Carebara* drm03 (CASENT0143975).

**Figure 7.**
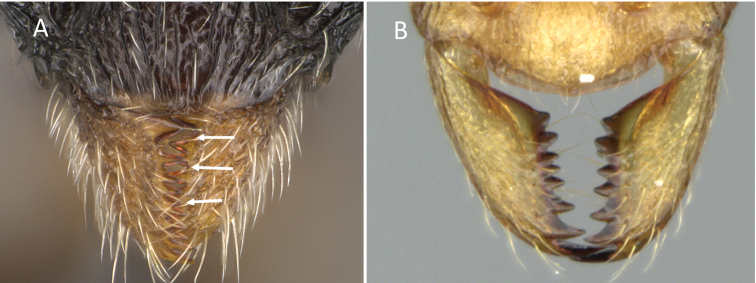
Mandible **A***Pilotrochusbesmerus* (CASENT0057183) **B***Malagidrissofina* (CASENT0906626).

**Figure 8.**
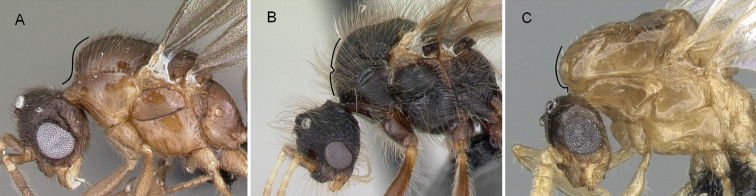
In profile view **A***Monomoriumtermitobium* (CASENT0135952) **B***Meranoplusmayri* (CASENT0062813) **C***Crematogasterhazolava* (CASENT0317643).

### ﻿Male-based key to genera of the tribe Solenopsidini in the Malagasy region

**Table:** 

1	Antennae 12-segmented	***Solenopsis***
–	Antennae 13-segmented	**2**
2	In full-face view, pedicel subglobular; posteromedian margin of clypeus effaced so that clypeus and frons form a continuous surface (Fig. 9A); mandible triangular with distinct basal angle, masticatory margin with exactly 4 teeth	***Erromyrma***
–	In full-face view, pedicel not globular, more cylindrical; posteromedian margin of clypeus visible (Fig. 9B); mandible spatulate to triangular, but its basal angle always indistinct, masticatory margin with 1 to 4 teeth	**3**
3	Forewing with five closed cells, 1m–cu cross-vein present (Fig. 10A). In profile, petiolar peduncle longer than postpetiolar length (Fig. 10C)	***Syllophopsis***
–	Forewing with four closed cells, 1m–cu cross-vein absent (Fig. 10B). In profile, petiolar peduncle absent or shorter than postpetiolar length (Fig. 10D)	**4**
4	With the head in full-face view, antennal scape short, barely reaching the posterior ocular margin; mandible long, curved, masticatory margin with 3 to 4 teeth (Fig. 11A)	***Monomorium***
–	With the head in full-face view, antennal scape long reaching the occipital margin; mandible short, spatulate, basal margin linear, unidentate (Fig. 11B)	***Adelomyrmex* (Seychelles)**

**Figure 9.**
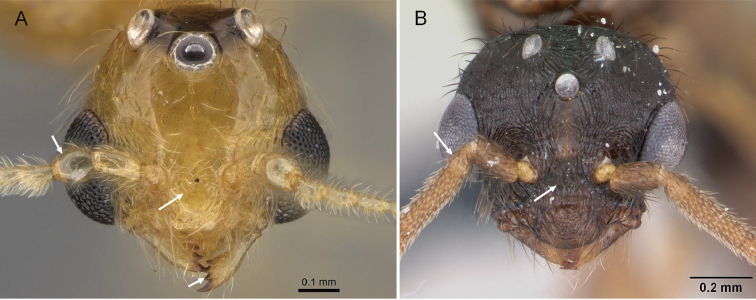
Head in full-face view showing the pedicel, mandible, postero-median margin of clypeus **A***Erromyrmalatinodis* (CASENT0788835) **B***Syllophopsiscryptobia* (CASENT0103340).

**Figure 10.**
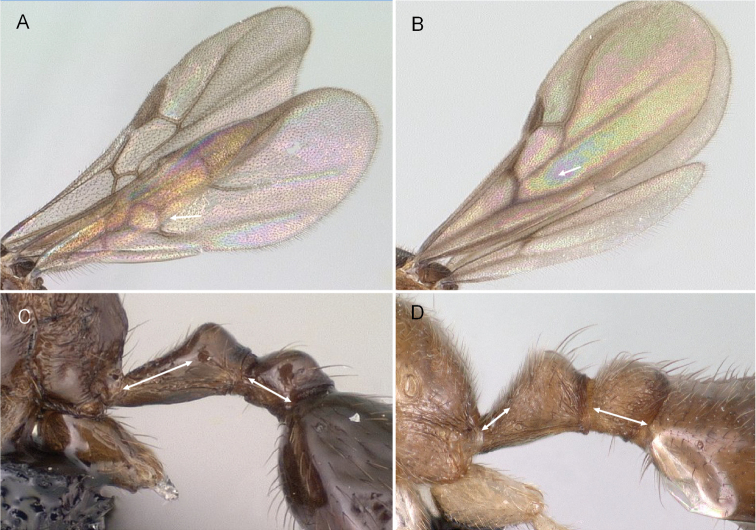
In profile view showing forewing, petiole and post petiole **A, C***Syllophopsismodesta* (CASENT0135642) **B***Monomoriumtermitobium* (CASENT0135673) **D***Monomoriumtermitobium* (CASENT0135952).

**Figure 11.**
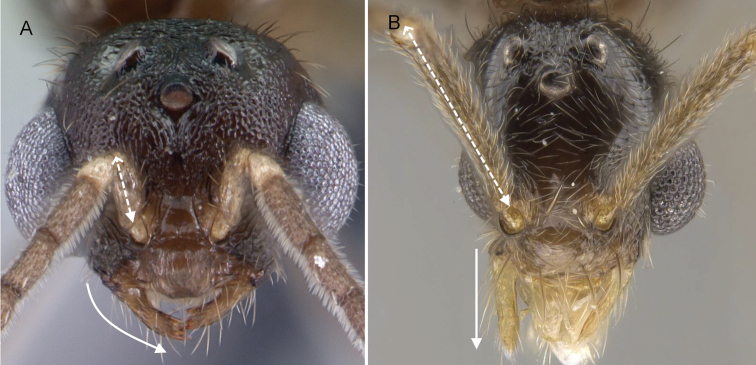
Head in full-face view showing mandible and scape **A***Monomoriumexiguum* (CASENT0135614) **B***Adelomyrmex* sc01 (CASENT0160764).

## ﻿Discussion

**Figure 12.**
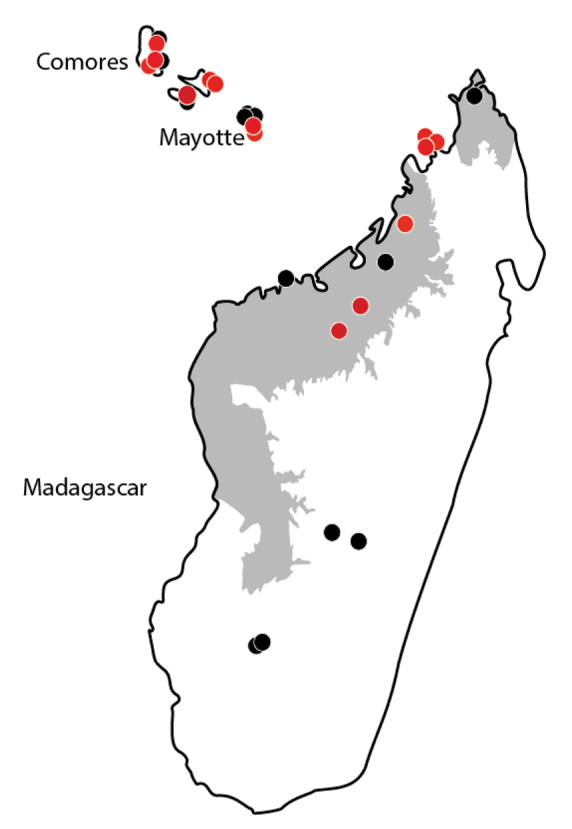
Distribution of *Erromyrmalatinodis* in the Malagasy region. Red indicates localities of sequenced specimens.

In the Malagasy region, *Erromyrmalatinodis* was collected from Comoros, Madagascar and Mayotte (see Fig. 12). The species inhabits montane rainforest, mangrove, *Uapaca* woodland, dry forest, and anthropogenic habitats from elevations of 2 to 1726 m. Workers were collected from a range of microhabitats and methods including foraging on low vegetation, on the ground, in ground nests, sifted litter, under stones, rotten wood or from dead twigs above ground. The males were collected along with workers and queens under the bark on the main trunk of a mango tree along a village road.

The males for this species were only collected after six expeditions. They are not collected by traditional means for example: malaise traps sampling or UV light samples from the region. Initial expeditions to known localities in northern Madagascar did not find the males. Colonies were kept alive for over a year without the production of males. Two males were finally found at one of the known collection sites.

The C01 data confirms the identification of the males and also shows a pattern of 0% sequence divergence between the samples from Madagascar, Comoros, and Mayotte. The lack of sequence divergence across island systems supports the hypothesis that this species is introduced in the region. Low sequence diversity could also be explained by other factors such as reproductive systems. The difficulty of finding males could be linked to a reproductive system that would reduce sequence divergence.

## Supplementary Material

XML Treatment for Erromyrma latinodis
